# Prognostic nutritional index and survival in prostate cancer: an updated systematic review and meta-analysis

**DOI:** 10.3389/fnut.2025.1736450

**Published:** 2026-01-27

**Authors:** Gang Wang, Qihang Wu, Telei Chen

**Affiliations:** Department of Urology, Yinzhou No. 2 Hospital, Ningbo, Zhejiang, China

**Keywords:** meta-analysis, PNI, prognostic nutritional index, prostate cancer, systematic review

## Abstract

**Objective:**

To evaluate the predictive value of the prognostic nutritional index (PNI) in prostate cancer patients. Compared with previous reviews, this study is the first to systematically grade and evaluate the quality of evidence regarding the association between PNI and prostate cancer prognosis using the Grading of Recommendations, Assessment, Development, and Evaluations (GRADE) framework, which can provide more reliable and transparent evidence for clinical practice.

**Methods:**

A systematic search was conducted across PubMed, Embase, Web of Science, and Cochrane databases up to June 2025. Study quality was assessed using the Newcastle–Ottawa Scale. Outcomes included associations between PNI and overall survival (OS) and progression-free survival (PFS). Meta-analysis, Egger's test, and sensitivity analysis were performed using Review Manager 5.4.1 and Stata 15.1. The certainty of evidence for each outcome was evaluated and graded according to GRADE.

**Results:**

The systematic search identified 857 related studies, with 11 studies included in the meta-analysis. The meta-analysis revealed that a lower PNI was significantly associated with shorter OS (hazard ratio (HR): 2.03; 95% confidence interval (CI): 1.68, 2.46; *P* < 0.00001) and PFS (HR: 2.03; 95% CI: 1.65, 2.50; *P* < 0.00001). There was no significant publication bias for OS (*P* = 0.051), but there was significant publication bias for PFS (*P* = 0.014). Sensitivity analyses confirmed that the results for OS and PFS were stable and reliable. Regarding the certainty of evidence, OS was rated as moderate quality evidence, while the PFS was rated as low quality.

**Conclusions:**

Low PNI is associated with shorter OS and PFS in prostate cancer patients. Considering the inherent limitations of this study, more prospective studies are needed to confirm the association between PNI and the prognosis of prostate cancer patients.

**Systematic review registration:**

https://www.crd.york.ac.uk/PROSPERO/view/CRD420251154118, identifier: CRD420251154118.

## 1 Introduction

As one of the most common malignant tumors in men worldwide, the incidence and mortality of prostate cancer have continued to increase over the past few decades, posing a major public health challenge, especially in aging societies (1, 2). In clinical practice, accurate risk stratification is of critical significance for the selection of treatment options such as active monitoring, radical surgery, radiotherapy or systemic treatment, and for prognostic judgment (3, 4). The current mainstream prognostic assessment tools mainly rely on pathological characteristics, serum prostate-specific antigen (PSA) levels, and clinical staging systems such as the TNM classification (5, 6). Although these indicators play a key role in disease diagnosis and initial stratification, they have significant limitations in reflecting the patient's overall physiological state, treatment tolerance, and long-term survival potential (7). Especially for metastatic patients receiving new endocrine therapies or chemotherapy such as abiraterone and enzalutamide, traditional indicators fail to fully reflect the heterogeneity of tumor biological behavior (5, 7, 8). This assessment gap has prompted researchers to continuously explore new prognostic markers that can integrate the body's systemic state.

In recent years, research on the tumor microenvironment has revealed the central role of the nutrition-inflammation axis in cancer progression (9, 10). The prognostic nutritional index (PNI) is a composite index calculated from serum albumin concentration and peripheral blood lymphocyte count. Because it can objectively reflect the body's immune defense capacity and nutritional reserve status, it has shown independent prognostic value in various solid tumors such as gastric cancer, colorectal cancer, and non-small cell lung cancer (11–14). Its theoretical basis is that hypoalbuminemia indicates protein metabolism disorders and a chronic inflammatory state, while lymphopenia reflects an impaired anti-tumor immune response; together, these conditions constitute a favorable microenvironment for tumor progression (15, 16). In the field of prostate cancer, preliminary studies have shown that PNI may be associated with disease progression, but research in this field is highly fragmented. On one hand, the existing evidence mainly comes from single-center, observational studies, with generally limited sample sizes and homogeneous population characteristics. On the other hand, there is significant heterogeneity in research design, including a lack of consensus on the determination of PNI cutoff values, inconsistent selection of prognostic endpoints, and varying degrees of control for confounding factors (17–20). These methodological differences make it difficult to directly compare the conclusions of different studies and may even lead to contradictory findings, which seriously hinders the scientific evaluation of the clinical applicability of PNI. In 2024, Tobing et al. (21) published a meta-analysis exploring the prognostic value of PNI for patients with prostate cancer. This meta-analysis included clinical studies before 2023, and the results suggested that low PNI values were associated with shorter overall survival (OS) and progression-free survival (PFS). Similarly, Zheng et al. (22) published a meta-analysis in 2024, including studies prior to March 2023. Their results showed that a low pretreatment PNI was significantly correlated with worse OS and PFS in patients with prostate cancer. However, the meta-analysis by Tobing et al. (21) and Zheng et al. (22) did not provide any evidence grading for the results, and new clinical studies have been published since its release (11, 17–19), and conclusions are not entirely consistent.

In order to address the above knowledge gaps and reduce evidence heterogeneity, this study was designed and implemented a systematic evidence-based integration work. The aim of this study is to comprehensively collect all clinical studies exploring the association between PNI and prostate cancer prognosis published up to June 2025 through systematic literature retrieval. The core objectives of the study focus on two key aspects: first, to determine whether low PNI can serve as an independent predictor of all-cause mortality risk in patients with prostate cancer; second, to standardize the quality of existing evidence using the GRADE framework in order to provide clear recommendations for clinical practice. This study aims to establish strict and unified inclusion criteria, adopt a two-investigator independent screening and data extraction process, and apply the Newcastle-Ottawa Scale to objectively evaluate the quality of the study. It is committed to producing highly credible synthesized evidence, ultimately providing a scientific foundation for optimizing the prostate cancer prognosis evaluation system and formulating personalized nutritional intervention strategies.

## 2 Methods

### 2.1 Literature search

This meta-analysis followed the PRISMA 2020 statement (23) and was prospectively registered in PROSPERO (CRD420251154118). A systematic literature search via PubMed, Embase, Web of Science, and Cochrane databases was conducted up to June 2025 to identify studies evaluating the prognostic value of PNI in prostate cancer. The searching terms used in Pubmed were: ((“Prostatic Neoplasms”[Mesh]) OR ((((((Prostatic Neoplasm) OR (Prostate Neoplasm)) OR (Prostate Cancer)) OR (Cancer of Prostate)) OR (Cancer of the Prostate)) OR (Prostatic Cancer))) AND ((prognostic nutritional index[Title/Abstract]) OR (PNI)). Additionally, the reference lists of all incorporated randomized controlled trials were manually scrutinized. There are no language or date restrictions for literature retrieval. Two researchers (GW and QHW) independently retrieved and evaluated relevant articles. Any discrepancies in literature identification were addressed through the third author (TLC). Searching details are provided in Supplementary Table S1.

### 2.2 Inclusion and exclusion criteria

Inclusion criteria:

P: Patients diagnosed with prostate cancer.

E: Low PNI (The formula for calculating PNI is: PNI = Serum albumin (g/dL) + 5 × Lymphocyte count (10∧9/L)).

C: High PNI.

O: Any survival outcome, including overall survival (OS), progression-free survival (PFS), etc.

S: Study design was cohort or case-control.

Exclusion criteria: study protocols, unpublished studies, non-original studies (including letters, comments, abstracts, correction, and reply), single-arm studies, studies without sufficient data (the hazard ratio (HR) and the corresponding 95% confidence intervals (CIs) for survival outcomes cannot be directly extracted or calculated from the available data), and reviews were excluded.

### 2.3 Data abstraction

Two investigators independently extracted data. Discrepancies were solved by the third author. Following information was abstracted from eligible studies: first author name, published year, study region, study design, study population, sample size, age, cut-off, OS, PFS. Corresponding authors were contacted to obtain missing data. In our meta-analysis, we prioritize extracting HRs that are adjusted for multivariates (such as age, Gleason score, PSA level, metastasis burden, and treatment type), and only use univariate HRs or estimate them from Kaplan–Meier curves when they are not available.

### 2.4 Quality evaluation

The quality of included cohort and case-control studies was assessed using the Newcastle-Ottawa Scale (NOS) (24), with studies scoring 7–9 points considered high quality (25). Two authors independently assessed all included studies' quality, and any disagreements were settled by discussion.

### 2.5 Statistical analysis

Data analysis was performed using Review Manager (RevMan) 5.4.1 (developed by the Cochrane Collaboration, software specifically for systematic reviews and meta-analyses). For survival data, HRs were used as the pooled effect size; for dichotomous data, odds ratios (ORs) were used. Each pooled effect size was reported with a point estimate and a 95% CI. Given that clinical and methodological heterogeneity was anticipated among included studies, all outcomes were calculated using a random-effects model (DerSimonian and Laird method). Heterogeneity of outcomes was assessed using the chi-square test (Cochrane's *Q* test) and the inconsistency index (*I*^2^ statistic). Significant heterogeneity was considered to exist if the *Q* test *P*-value was < 0.10 or *I*^2^ > 50% (26, 27). For outcomes involving more than two studies, sensitivity analysis was performed by eliminating individual studies one by one to assess the impact of each study on the stability of the pooled outcome. Stata 15.1 software was used to assess potential publication bias using Egger's regression test. A *P*-value < 0.05 was considered statistically significant (28). Furthermore, the quality of evidence for each outcome was assessed and graded as “high,” “moderate,” “low,” or “very low” according to the GRADE (Grading of Recommendations, Assessment, Development, and Evaluations) framework (29).

## 3 Results

### 3.1 Literature retrieval, study characteristics, and baseline

**Figure 1:**
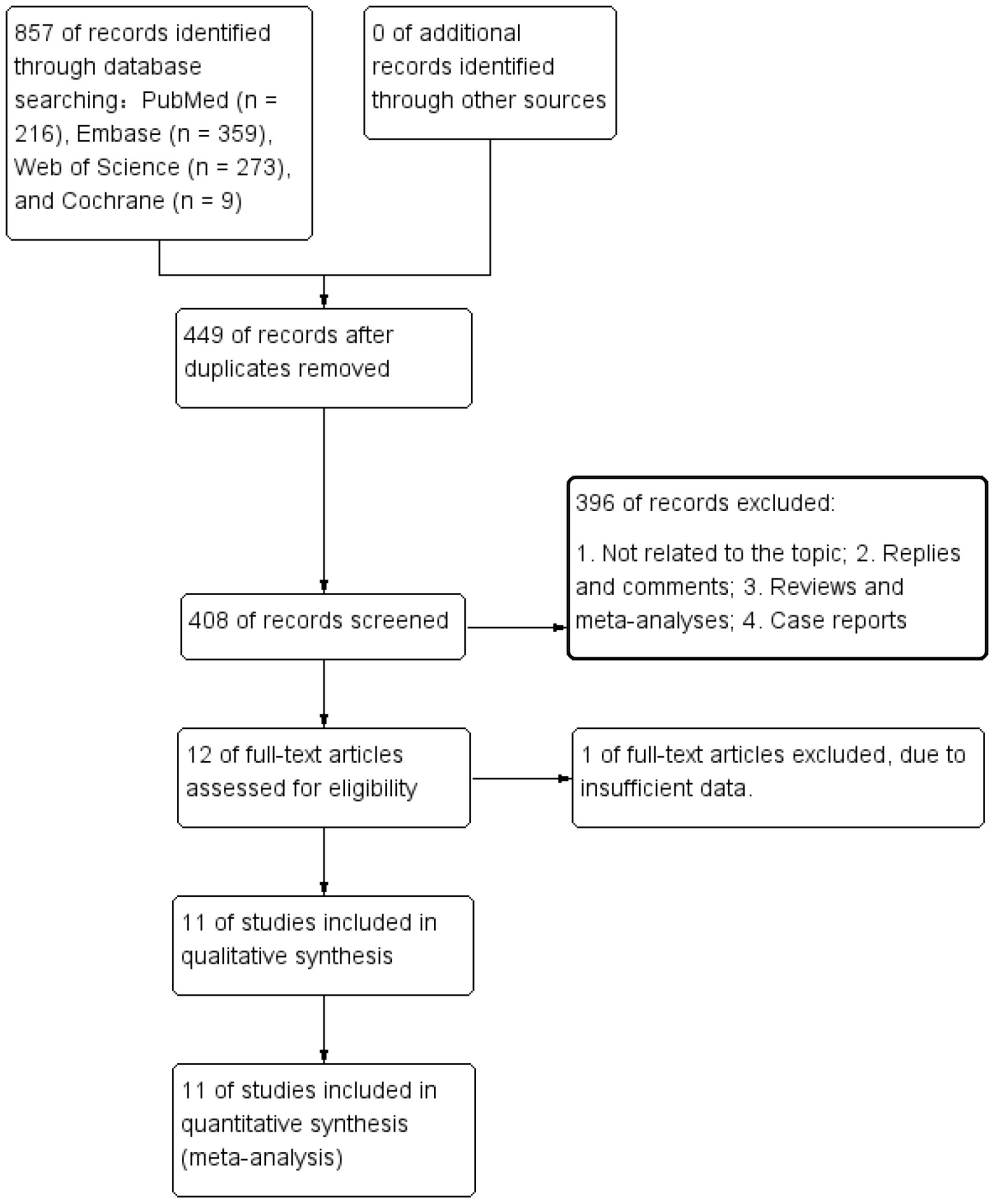
Flowchart of the systematic search and selection process.

The literature retrieval and selection process flowchart is shown in Figure 1. A systematic literature search identified 857 related studies in PubMed (*n* = 216), Embase (*n* = 359), Web of Science (*n* = 273), and Cochrane (*n* = 9). After removing duplicates, 408 titles and abstracts were evaluated. Ultimately, 11 studies including 2,597 patients were included for meta-analysis (17–20, 30–36). Some of the studies involved multiple parallel cohorts, resulting in a total of 12 comparative groups extracted. Table 1 presents the characteristics and quality assessment of each eligible study. The included literature was published between 2017 and 2025.

**Table 1:** Characteristics of eligible studies and assessment of risk of bias.

**Study**	**Country**	**Study design**	**Population**	**No. of patients**	**Mean/ median age**	**Mean/ median PSA**	**PNI cut-off**	**NOS score**
Ellez 2023	Turkey	Retrospective cohort	Metastatic castration-sensitive prostate carcinoma	108	NA	NA	49.75	8
Fan 2017	China	Retrospective cohort	Metastatic castration-resistant prostate cancer patients treated with abiraterone	112	72	63.4	50.5	9
Hacioglu 2025	Turkey	Retrospective cohort	Metastatic hormone-sensitive prostate cancer	167	NA	NA	49.98	8
Kucukarda 2022	Turkey	Retrospective cohort	Metastatic castration-resistant prostate cancer treated with abiraterone acetate or enzalutamide	101	71	66.9	46.62	7
Li 2020	China	Retrospective cohort	Patients undergoing androgen deprivation therapy	280	76	91.2	50.2	8
Li 2022	China	Retrospective cohort	Patients treated with robot-assisted laparoscopic radical prostatectomy	136	NA	15.39	46.03	7
Li 2024a	China	Retrospective cohort	Elderly patients with prostate cancer	384	71.35	NA	Moderate malnutrition	7
Li 2024b	China	Retrospective cohort	Elderly patients with prostate cancer	384	71.35	NA	Severe malnutrition	7
Shu 2018	China	Retrospective cohort	Patients with high-risk localized prostate cancer who underwent radical prostatectomy	440	NA	NA	50.5	7
Taban 2025	Turkey	Retrospective cohort	Prostate Cancer Treated with Abiraterone, Enzalutamide or Cabazitaxel	299	65.2	58.5	40.8	7
Yamada 2023	Japan	Retrospective cohort	Patients with metastatic hormone-sensitive prostate cancer	353	73	266.18	47.71	7
Yang 2022	China	Retrospective cohort	Chinese patients with high/extremely high-risk prostate cancer after radical prostatectomy	193	NA	NA	46.23	9

a and b represent two research groups from the same study.

### 3.2 Prognostic value of PNI for OS

**Figure 2:**
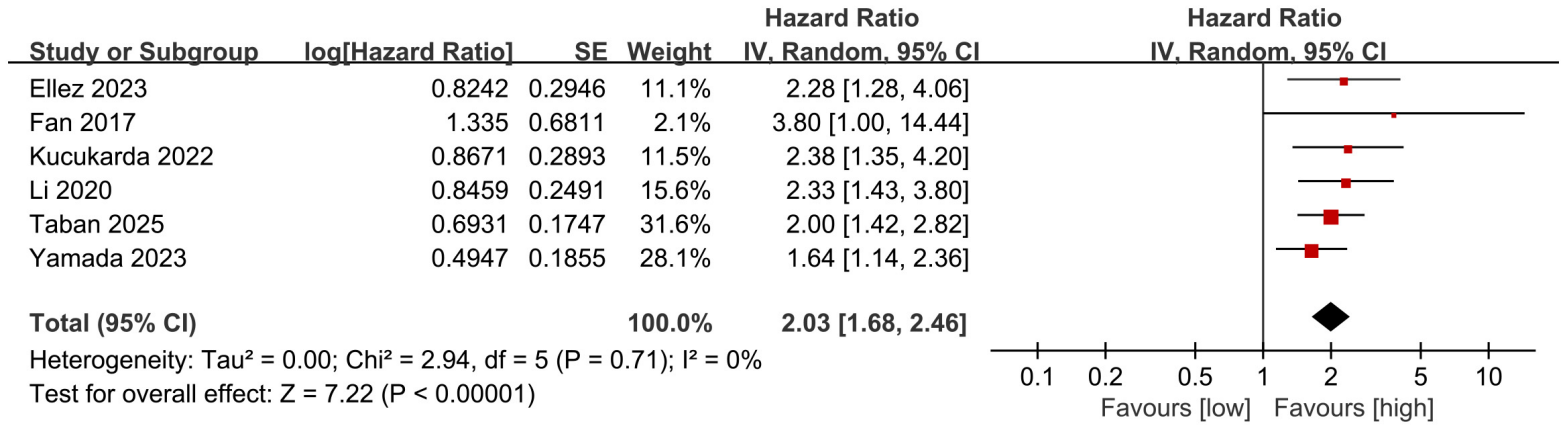
Forest plots of the prognostic value of PNI for OS.

OS results were synthesized from 6 comparative groups. Meta-analysis revealed that a lower PNI was significantly associated with a shorter OS (HR: 2.03; 95% CI: 1.68, 2.46; *P* < 0.00001), without significant heterogeneity (*I*^2^ = 0%, *P* = 0.71) (Figure 2).

### 3.3 Prognostic value of PNI for PFS

**Figure 3:**
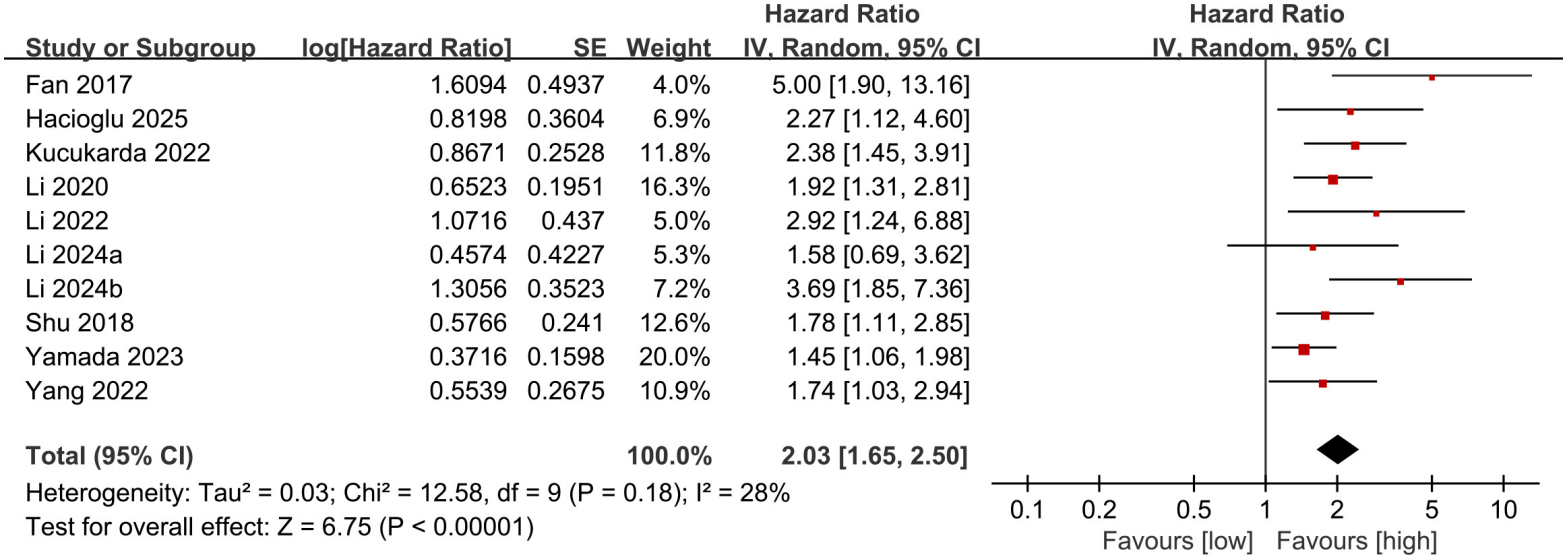
Forest plots of the prognostic value of PNI for PFS.

PFS results were synthesized from 10 comparative groups. Meta-analysis revealed that a lower PNI was significantly associated with a shorter PFS (HR: 2.03; 95% CI: 1.65, 2.50; *P* < 0.00001), without significant heterogeneity (*I*^2^ = 28%, *P* = 0.18) (Figure 3).

### 3.4 Publication bias

**Figure 4:**
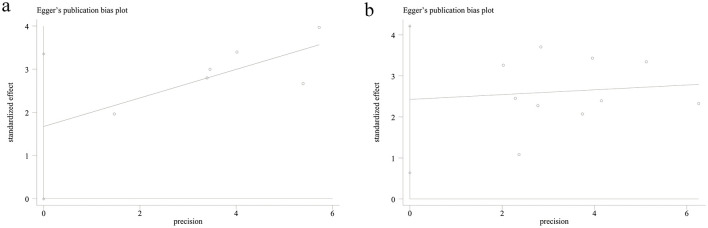
Egger's publication bias plot of the prognostic value of PNI for **(a)** OS and **(b)** PFS.

The potential publication bias for the prognostic value of PNI for OS and PFS was assessed using Egger's regression tests. It was found that there was no significant publication bias for OS (*P* = 0.051, Figure 4a), but there was significant publication bias for PFS (*P* = 0.014, Figure 4b).

### 3.5 Sensitivity analysis

**Figure 5:**
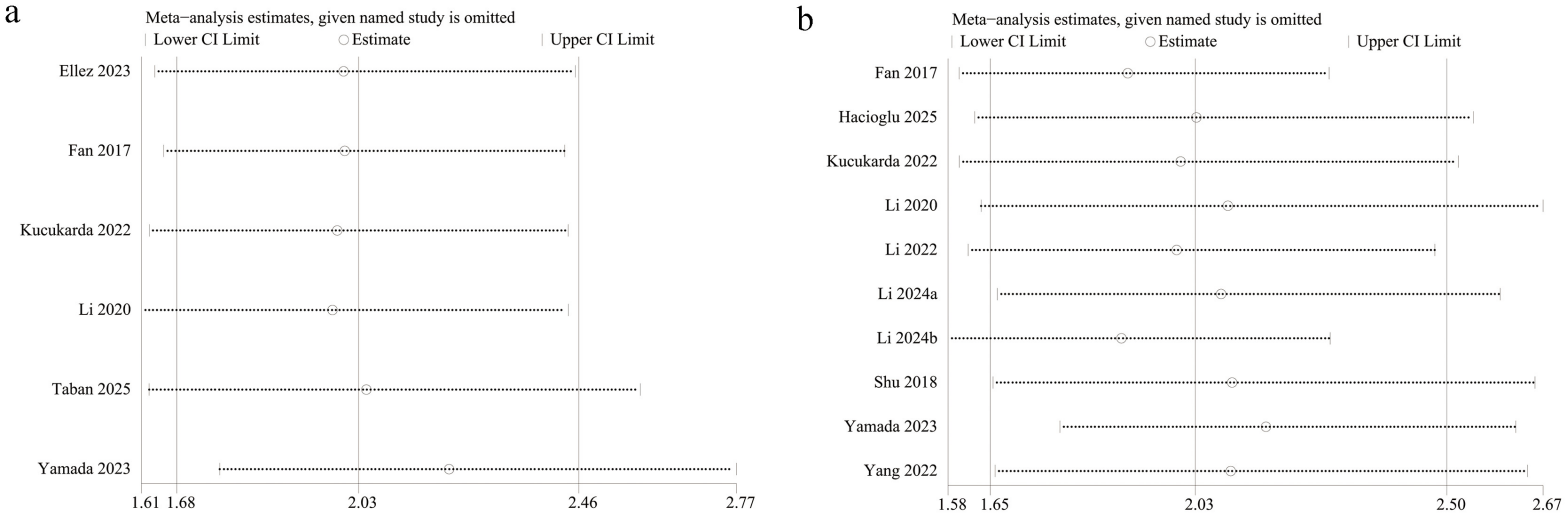
Sensitivity analysis of the prognostic value of PNI for **(a)** OS and **(b)** PFS.

A sensitivity analysis was performed to assess each study's effect on the total HR for the prognostic value of PNI for OS and PFS by excluding eligible studies one by one. The new total HR remained stable after removing each study for OS (Figure 5a) and PFS (Figure 5b).

### 3.6 GRADE rating

The assessment of evidence quality using the GRADE framework revealed discrepancies in the quality of evidence regarding the association between PNI and different survival outcomes in prostate cancer patients. For OS, the quality of evidence was rated as moderate. This was based on the fact that the meta-analysis showed a significant association between low PNI and shorter OS (HR = 2.03, 95% CI: 1.68–2.46), with high precision (narrow confidence interval) and very low heterogeneity (*I*^2^ = 0%). Although all included studies were observational designs, we upgraded this due to the large observed effect size (HR > 2). Furthermore, risk assessment did not identify any serious risks of bias, indirectness, or imprecision. Although the *P*-value of Egger's test (0.051) was on the borderline of statistical significance, we determined that it did not indicate clear publication bias and therefore did not downgrade the evidence for OS. For PFS, the quality of evidence was rated as low. Although the pooled HR was also 2.03 (95% CI: 1.65–2.50), with low heterogeneity (*I*^2^ = 28%) and no serious problems found in other domains (such as risk of bias and imprecision), Egger's test detected significant publication bias (*P* = 0.014), leading to a downgrade of the level of evidence. This result suggests that published studies on PNI and PFS may carry the risk of selectively reporting positive results, thus affecting the reliability of the findings (Table 2).

**Table 2:** GRADE rating of outcome.

**No. of groups**	**Outcomes**	**HR**	**95%CI**	***I*^2^; *P* value**	**Risk of bias**	**Inconsistency**	**Indirectness**	**Imprecision**	**Publication bias**	**Plausible con- founding**	**Magnitude of effect**	**Dose-response gradient**	**GRADE**
6	OS	2.03	1.68, 2.46	0%; *P* = 0.71	No serious risk	No serious inconsistency	No serious indirectness	No serious imprecision	Undetected	Would not reduce effect	Yes	No	Moderate
10	PFS	2.03	1.65, 2.50	28%; *P* = 0.18	No serious risk	No serious inconsistency	No serious indirectness	No serious imprecision	Strongly suspected	Would not reduce effect	Yes	No	Low

## 4 Discussion

This study systematically integrated data from 11 clinical studies on prostate cancer patients and updated and confirmed the universal value of PNI as a predictor of survival in prostate cancer. The core findings showed that low PNI status was significantly associated with shorter OS and had similar efficacy in predicting PFS. This result has potential clinical significance—compared with traditional indicators such as Gleason grade or PSA level, PNI provides a supplementary dimension for risk stratification across treatment modalities, from radical surgery to new endocrine therapies, by quantifying the body's nutritional-immune status (21, 22). It is particularly noteworthy that both primary endpoints showed extremely low heterogeneity, suggesting that the prognostic efficacy of PNI is consistent across different geographical populations, disease stages and treatment backgrounds. This finding partially explains the contradictory conclusions of early single-center studies: for example, Hacioglu 2025's study (18) reported that PNI had an HR of 2.27 for PFS, while Li's 2024 study (19) did not find a significant association between PNI and PFS. However, through a large sample combined analysis, this meta-analysis confirmed that this difference is more likely due to insufficient statistical power rather than fundamental biological differences. Further analysis of the source of this robustness reveals that the core advantage of PNI lies in the universality of its biological basis: as an integrated indicator of systemic inflammation and nutritional status, it is not limited to the function of a single organ or local tumor characteristics, but reflects the overall host-tumor interaction (37, 38). This global feature enables PNI to transcend the limitations of traditional staging systems.

Compared with the meta-analyses by Tobing et al. (21) and Zheng et al. (22), the innovations of this study are: (1) the inclusion of more updated and comprehensive research data up to 2025, which improves statistical power and may reduce publication bias; (2) the first systematic introduction and application of the GRADE evidence grading criteria in the field of prostate cancer PNI prognosis research, which standardizes the assessment of the reliability of the evidence; and (3) the implementation of more rigorous publication bias tests (such as Egger's test) and sensitivity analyses to ensure the robustness of the pooled results. These additions enhance the incremental contribution of this study compared to previous studies.

In-depth analysis of heterogeneity sources is key to understanding the generalizability of the conclusions. Although the overall heterogeneity of the primary endpoint was low, there are still methodological differences among the included studies that need to be discussed. The primary difference lies in the determination of the PNI cutoff value: as shown in Table 1, the cutoff values used ranged from 40.8 to 50.5. This difference arises from differences in study populations and statistical approaches. Some studies determined the optimal cutoff using ROC curves, while others used the median. Importantly, sensitivity analysis confirmed that the overall HR remained stable regardless of which study was excluded, indicating that differences in cutoff values did not substantially impact the overall conclusions. Secondly, variability in treatment backgrounds warrants special consideration: Taban et al. (17) and Ellez et al. (30) focused on the responses to abiraterone or enzalutamide in patients with mCRPC, while Yang et al. (36) and Li et al. (34) analyzed the prognosis of radical surgery. Finally, differences in follow-up duration may affect the accumulation of endpoint events, but Cochran's *Q* test showed that the time factor did not introduce significant bias. It is worth exploring that this heterogeneity control ability may be due to the unique properties of the PNI indicator: as a continuous variable, it has a linear relationship with the prediction of prognosis, so even if the threshold is set differently, its risk trend can remain consistent (39). This contrasts sharply with binary variables, which are highly sensitive to the choice of cut-off points.

Although sensitivity analysis confirmed the robustness of the pooled HR values, differences in clinical context may affect the generalizability of our results. This means that caution should be exercised when directly applying the general conclusion that “lower PNI predicts worse survival” to a specific clinical subgroup (e.g., only mCRPC patients), as the effect size may vary depending on the treatment environment and disease biology. Therefore, the current pooled estimate based on the entire population should be considered as general evidence of association. Given the relatively limited number of studies included in this study (11), statistical subgroup analyses based on disease stage or treatment type are not powerful enough. We strongly recommend that future studies, with more original research, conduct such subgroup analyses or individual patient data (IPD) meta-analyses to clarify the precise predictive value of PNI in different clinical scenarios (e.g., localized disease vs. metastatic disease, mHSPC vs. mCRPC, different first-line treatment regimens), which will greatly promote the clinical application of PNI in precision medicine.

The evaluation of publication bias revealed potential vulnerabilities in the body of evidence. Egger's regression test showed that no significant publication bias was detected in the OS analysis, however there was a clear risk of bias in the PFS analysis. This discrepancy may be due to three factors. First, the tendency for positive findings to be preferentially published is more pronounced in studies focusing on surrogate endpoints. As an early indicator of tumor progression, PFS is more likely to yield publishable positive results, while OS, which requires longer follow-up, may lead to early termination of studies with negative or null results due to resource constraints (40, 41). Second, among the 10 included PFS studies, the effect size of small sample studies [such as Fan et al. (31)] was significantly higher than that of large sample studies [such as Yamada et al. (20)], which is consistent with the bias feature of “small sample effect”. Although the sensitivity analysis confirmed the robustness of the PFS results, the GRADE system still downgraded its evidence quality to “low”, suggesting that further verification through prospective registration studies is needed in the future. This finding has important implications for evidence-based practice: if current clinical guidelines recommend the use of PNI based solely on PFS evidence, its actual effectiveness may be overestimated. Therefore, we strongly recommend that when developing PNI-related clinical pathways, OS evidence should be relied upon first, while PFS-oriented decisions should be supplemented by other confirmatory indicators.

As a composite indicator of serum albumin and lymphocyte count, the biological basis of PNI's predictive efficacy may involve three pathways. First, hypoalbuminemia directly reflects the state of protein-energy malnutrition, resulting in decreased tissue repair capacity and reduced treatment tolerance—this is particularly prominent in mCRPC patients receiving cytotoxic drugs such as abiraterone, and the risk of dose adjustment is significantly increased in malnourished patients (42, 43). The underlying mechanism involves albumin's role as the main drug-carrying protein, reduced albumin levels lead to a higher proportion of unbound (free) drug in the bloodstream, thereby enhancing the risk of toxic side effects (44). Second, lymphocytopenia indicates systemic hyperinflammatory response and impaired immune surveillance: tumor-associated macrophages can release proinflammatory factors such as IL-6 to inhibit lymphocyte proliferation, while inducing reduced albumin synthesis, forming a vicious cycle of “malnutrition-inflammation-immunosuppression” (45, 46). This cycle is significantly amplified in the microenvironment of prostate cancer bone metastasis—TGF-β released during osteoclast activation can further inhibit CD8+T cell function. Second, PNI may indirectly reflect the characteristics of tumor metabolic reprogramming: prostate cancer cells competitively consume blood amino acid reserves through glutamine decomposition, exacerbating hypoalbuminemia (47); at the same time, factors such as TGF-β released by the tumor microenvironment can inhibit lymphocyte infiltration. Together, these mechanisms constitute a positive feedback loop: malnutrition weakens immune response → immune escape promotes tumor progression → increased tumor load further consumes nutritional reserves.

According to the GRADE assessment, the evidence for PNI's prediction of OS was rated as “moderate quality”, mainly due to its significant effect size (HR > 2) and precise results (narrow 95% CI). In contrast, the evidence for PFS was rated as “low quality”, mainly due to significant publication bias. This suggests that PNI should be applied in a stratified manner in clinical practice: in the OS prediction scenario, PNI can be used as a reference indicator for survival benefit; however, for PFS-related decisions, it is necessary to carefully combine traditional indicators for comprehensive judgment. The specific implementation framework can be outlined as follows: For patients with newly diagnosed localized cancer, preoperative PNI can be used as a reference indicator for the initiation of adjuvant therapy. In patients undergoing new endocrine therapy, low PNI before treatment indicates the need for more intensive toxicity monitoring and nutritional support. It is worth noting that PNI is inexpensive to detect and can replace expensive molecular tests in resource-limited areas. However, current evidence is not sufficient to support the inclusion of PNI in the formal prognostic scoring system. The key limitation is the lack of dynamic monitoring data, and all included studies have not systematically analyzed the trajectory of PNI changes (48). Therefore, we recommend that before implementing PNI hierarchical management, a standardized PNI monitoring module should be established in the electronic medical record system to accumulate time series data for future model optimization.

Furthermore, the malnutrition and systemic inflammatory state indicated by low PNI may be an area for intervention, including: (1) nutritional support: for example, targeted high-protein and branched-chain amino acid supplementation may help improve nutritional status, thereby potentially affecting PNI levels and tumor prognosis; (2) anti-inflammatory intervention: possible management strategies for the chronic inflammatory state reflected by PNI; (3) exercise therapy: the potential value of regular exercise as a non-pharmacological intervention to improve the body's inflammatory state and muscle mass. These intervention proposals still require prospective clinical studies to verify whether they can improve patient prognosis by improving PNI.

This study has several limitations. First, all included studies were observational in design, which raises the possibility of residual confounding. Although most studies used multivariate Cox regression to adjust basic variables, the correction of molecular features such as PTEN loss and BRCA mutation was generally missing. The absence of adjustment for these factors may have led to an overestimation of the observed effect size. Second, the calculation of PNI only relies on a single baseline test and cannot reflect the dynamic changes in nutritional status during treatment. More importantly, due to the lack of original data, this meta-analysis cannot answer key questions such as “differences in the predictive efficacy of PNI in patients of different ages” or “optimization of cutoff values for different races” through subgroup analysis. Based on this, future studies should focus on three key advancements: first, design a prospective dynamic monitoring cohort, systematically collect PNI data before, during and after treatment, and establish a prediction model based on the PNI change rate; second, conduct treatment response-oriented subgroup analysis to clarify the predictive value of PNI in a specific drug treatment population; third, explore the combination model of PNI with new immune indicators such as platelet-lymphocyte ratio and systemic immune inflammatory index, and improve the accuracy of risk stratification through multi-parameter integration. The ultimate goal is to integrate PNI into the precision medicine framework for prostate cancer, enabling a translational leap from prognostic prediction to actionable intervention targets—for instance, implementing personalized nutritional interventions or immunomodulatory therapies for patients with low PNI and systematically evaluating their impact on survival outcomes.

## 5 Conclusion

This study systematically meta-analyzed 11 clinical studies and confirmed that low PNI is significantly associated with poorer survival outcomes in patients with prostate cancer. According to the GRADE assessment, the evidence supporting the association with overall survival (OS) was of moderate quality, while the evidence for progression-free survival (PFS) was rated as low quality due to significant publication bias. However, considering the limitations of this study, such as the fact that most of the studies were retrospective, lack of dynamic monitoring data during PNI treatment, and subgroup analysis data, prospective cohort studies are needed. These studies should systematically collect longitudinal PNI data and explore its interaction with tumor molecular characteristics to facilitate the translational application of PNI from prognostic prediction to actionable clinical intervention targets.

## Data Availability

The original contributions presented in the study are included in the article/supplementary material, further inquiries can be directed to the corresponding author.
