# Supplementary material for: Prognostic nutritional index and survival in prostate cancer: an updated systematic review and meta-analysis
Source: Front Nutr. 2026 Jan 27;12:1736450. doi: 10.3389/fnut.2025.1736450 (PMC12886034; doi:10.3389/fnut.2025.1736450)
Supplement: Supplementary file 1 [file Data_Sheet_1.docx]

TableS1 Literature search strategy

Pubmed-216

(("Prostatic Neoplasms"[Mesh]) OR ((((((Prostatic Neoplasm) OR (Prostate Neoplasm)) OR (Prostate Cancer)) OR (Cancer of Prostate)) OR (Cancer of the Prostate)) OR (Prostatic Cancer))) AND ((prognostic nutritional index[Title/Abstract]) OR (PNI))

Embase-359

((Prostatic Neoplasms or (Prostatic Neoplasm or Prostate Neoplasm or Prostate Cancer or Cancer of Prostate or Cancer of the Prostate or Prostatic Cancer)) and (prognostic nutritional index or PNI)).af.

Cochrane-9

((Prostatic Neoplasms or (Prostatic Neoplasm or Prostate Neoplasm or Prostate Cancer or Cancer of Prostate or Cancer of the Prostate or Prostatic Cancer)) and (prognostic nutritional index or PNI)).af.

WOS-273

((Prostatic Neoplasms) OR ((((((Prostatic Neoplasm) OR (Prostate Neoplasm)) OR (Prostate Cancer)) OR (Cancer of Prostate)) OR (Cancer of the Prostate)) OR (Prostatic Cancer))) AND ((prognostic nutritional index) OR (PNI)) (All Fields)
